# Experiences and perspectives on pregnancy and motherhood in elite athletes – a qualitative study

**DOI:** 10.1080/26410397.2025.2501832

**Published:** 2025-05-07

**Authors:** Marlene Zahl Marken, Emilie Frederikke Mass Dalhaug, Lone Friis Thing, Frank Eirik Abrahamsen, Kari Bø, Lene Annette Hagen Haakstad

**Affiliations:** a Physical Therapist, Department of Sports Medicine, Norwegian School of Sports Sciences, Oslo, Norway.; b PhD Research Fellow, Department of Sports Medicine, Norwegian School of Sports Sciences, Oslo, Norway; c Professor and Head of Department, Department of Sport and Social Sciences, Norwegian School of Sports Sciences, Oslo, Norway; d Associate Professor, Department of Sport and Social Sciences, Norwegian School of Sports Sciences, Oslo, Norway; e Professor, Department of Sports Medicine, Norwegian School of Sports Sciences, Oslo, Norway; Research Group Leader, Department of Obstetrics and Gynecology, Akershus University Hospital, Oslo, Norway; f Professor, Department of Sports Medicine, Norwegian School of Sports Sciences, Oslo, Norway

**Keywords:** elite sports, pregnancy, postpartum, training, motherhood

## Abstract

Elite athletes routinely undertake strenuous training routines, which often involve high-intensity sessions. However, there are knowledge gaps in how they experience training during pregnancy and subsequent return to sport. Combined with inadequate financial and contractual safety, female athletes may jeopardise their careers when starting families. This study aimed to describe female athletes’ experiences and perspectives related to pregnancy and motherhood within the context of elite sports in Norway. We interviewed five world-class athletes between October 2022 and April 2023, using a descriptive qualitative approach. Full interview transcripts were analysed based on a reflexive thematic analysis model. Five overarching themes were identified: (1) uncertainty, (2) lack of supportive networks, (3) physical capacity, (4) the impact of postpartum return to sports on maternal health, and (5) combining motherhood and elite sports. Our findings emphasise the challenges that Norwegian pregnant and postpartum athletes face in balancing motherhood with successful careers, highlighting the importance of providing adequate support systems to ensure their health and the well-being of their child.

## Plain language summary

Elite athletes often face challenges when training during pregnancy, and returning to sports afterward. This study aimed to understand these challenges and the support the female athletes receive. We interviewed five top athletes from Norway and found five key points. (1) Athletes were unsure about safe training during and after pregnancy. This was because their coaches and organisations didn’t provide clear guidance. It led to stress about their performance, health, and their baby’s well-being. (2) Support from partners helped athletes manage both motherhood and their sports careers. (3) Many athletes felt strong and continued intense training until they gave birth. (4) Returning to sports after childbirth was tough without training guidelines. Some athletes also found it hard to maintain a healthy weight while breastfeeding. (5) Motherhood *sometimes* improved their performance and helped them return to sports. This study concludes there should be better support for pregnant and postpartum athletes in Norway. This would help them stay healthy, care for their children, and continue their sports careers successfully.

## Résumé

Les athlètes de haut niveau suivent régulièrement des entraînements éprouvants, avec des séances souvent extrêmement intenses. Néanmoins, il existe un manque de connaissance sur la manière dont les femmes vivent l’entraînement pendant la grossesse et la reprise du sport. Déjà touchées par une sécurité financière et contractuelle insuffisante, les athlètes féminines risquent de compromettre leur carrière lorsqu’elles fondent une famille. Cette étude visait à décrire les expériences et les points de vue des athlètes féminines concernant la grossesse et la maternité dans le contexte des sports de haut niveau en Norvège. Nous avons interrogé cinq sportives de renommée internationale, en utilisant une approche qualitative descriptive. Les transcriptions intégrales des entretiens ont été analysées selon un modèle d’analyse thématique réflexive. Cinq thème principaux ont été identifiés: (1) l’incertitude; (2) le manque de réseaux de soutien; (3) la capacité physique; (4) l’impact de la reprise du sport après l’accouchement sur la santé maternelle; et (5) la conciliation de la maternité et du sport de haut niveau. Nos résultats soulignent les difficultés rencontrées par les athlètes norvégiennes enceintes et en postpartum pour combiner maternité et réussite professionnelle, mettant en lumière l’importance de la mise en place de systèmes de soutien adéquats pour garantir leur santé et le bien-être de leur enfant.

## Resumen

Los atletas de alto rendimiento rutinariamente realizan rutinas de entrenamiento exigentes, que a menudo implican sesiones de alta intensidad. Sin embargo, existen brechas de conocimiento sobre su experiencia de entrenamiento durante el embarazo y su regreso posterior al deporte. Cuando esto se combina con una seguridad financiera y contractual inadecuada, las mujeres atletas podrían arriesgar su carrera profesional al iniciar una familia. Este estudio se propuso describir las experiencias y perspectivas de mujeres atletas relativas al embarazo y la maternidad en el contexto de deportes de alto rendimiento en Noruega. Entrevistamos a cinco atletas de clase mundial, utilizando un enfoque cualitativo descriptivo. Analizamos las transcripciones de entrevistas completas basándonos en un modelo de análisis temático reflexivo. Identificamos cinco temas generales: (1) incertidumbre, (2) la falta de redes de apoyo, (3) capacidad física, (4) el impacto que tiene el regreso posparto a los deportes en la salud materna y (5) la combinación de la maternidad y los deportes de alto rendimiento. Nuestros hallazgos hacen hincapié en los retos que las atletas noruegas embarazadas y posparto enfrentan para equilibrar la maternidad con una carrera profesional exitosa, y destacan la importancia de brindar sistemas de apoyo adecuado para garantizar su salud y el bienestar de su hijo/a.

## Introduction

The number of female athletes competing in elite sports has increased considerably worldwide over the past 30 years, and they now make up almost 50% of Olympic participants.^1^ The International Olympic Committee (IOC) and many of the national sports federations encourage female athletes to strive for high-level performance and state that they aim to support the health and well-being of their athletes.^1^ Female athletes who compete through their 30s are at the height of their careers when they may want to start a family.^2–5^ As a result, they navigate a rocky landscape while planning for pregnancy when participating in elite sports and are often influenced by the media voicing their opinions, stigmatisation, and the public’s conservative views on motherhood.^3,6–8^ Historically, women have been seen as weak and as having limited physical and mental capacity.^9^ They were therefore required to become sedentary during pregnancy and in the postpartum period to avoid endangering their baby or themselves. As western society has matured and activity has become a greater part of our daily life, social narratives that pregnant women should adopt a more cautious approach to their training routines and that they are inherently fragile during pregnancy and postpartum have persisted. However, in recent years these narratives are gradually being challenged by successful stories of female elite athletes managing pregnancy and motherhood while participating in training regimens at a high level.^3,10–12^ While research supports the safety and benefits of being physically active during pregnancy, the findings are mostly based on the general population.^11–15^

Elite athletes typically engage in rigorous training regimens that involve high-intensity training to reach or maintain athletic goals.^14^ However, our understanding of how to modify training routines during pregnancy to account for physiological changes and safeguard both mother and fetus remains significantly limited.^15–18^ Additionally, there is limited research on the optimal strategies for postpartum athletes to successfully return to sports while minimising the risk of injury and ensuring sports performance.^19–24^ Athletes report receiving scarce information and advice from coaches, clubs, and sports federations who often leave the responsibility up to the individual athlete to train themselves during pregnancy and postpartum.^12,23,25^ Meanwhile, numerous elite athletes challenge common perceptions about childbirth’s impact on athletic performance by consistently training at high intensities and heavy loads during pregnancy, and competing at their pre-pregnancy level upon return to sports.^5,11,12,14^

Qualitative studies show that varying systems of support, as well as financial security, heavily influence female elite athletes’ decisions to have children.^3,4,20,25–27^ Athletes often rely on achievements and competition results to maintain sponsorships and contracts with clubs.^3,4^ Moreover, they report feeling a lack of support by corporate sponsors or athletic governing bodies when planning pregnancy, during pregnancy, or in the postpartum period.^3,20^ This results in pregnancy being seen as a risk that may jeopardise their careers.^3,10,20,25,28^ Davenport and colleagues reported that athletes wished they had more time to recover postpartum before returning to sports, as changes to the body, both physically and mentally, are prominent.^10^ However, the pressure of resuming sports is often dictated by clubs, as well as by the household’s financial situation.^6,8,10,27–30^ One overarching theme found in the literature was pressure to embody the cultural stereotype of a “good mother”^2,7,31,32^ who prioritises their family’s needs over their own pursuits. The elite athletes straddle meeting the expectations of the “good mother” while also safeguarding their identity as a sportswoman.^2,3,6,8,14,28^ The experiences and perceptions of female elite athletes are strongly shaped by the political, societal, and cultural contexts of the countries in which studies are conducted. Hence, despite the progress in qualitative research, particularly in countries such as the US, Canada, Australia, and New Zealand,^2–4,6,8,10,20,25,26,28–30,33^ there is still a notable gap in understanding these themes on a global scale, particularly concerning female athletes from diverse cultural backgrounds. Among the Scandinavian countries, our literature review revealed only one study from Sweden that parallels our methodology; however, this study focuses exclusively on cross-country skiing and included participants working in both Norway and Sweden.^2^ In addition, the research differs in whether the studies have used a more comprehensive approach, engaging a larger participant pool and covering various aspects of pregnancy and maternity in elite sports,^3,10^ or if the focus has been on one individual athlete or singular topic such as breastfeeding.^4,6,8,26–30,33–35^

Through an extensive review of existing international research, we have identified a pressing need for comprehensive studies that cover a broad array of themes pertinent to pregnancy and motherhood in elite sports. These studies should incorporate different sports disciplines, span various age groups, and examine the availability and impact of supportive networks. As there is scant research involving Norwegian athletes, our study, therefore, aimed to gain a deeper understanding of the challenges faced by female athletes in Norway. Investigating how they balance elite sport, pregnancy, and family life, highlighting topics like health, training, competition, and practical challenges, may support future expectant athlete mothers to navigate this territory in Norway and on a global scale.

## Methods

We applied the “Eight Big Tent Criteria for Qualitative Quality.”^36^

### Study design and participant selection

Qualitative descriptive studies are often used when clear descriptions of phenomena are wanted.^37^ Using descriptive thematic analysis with a reflexive and inductive alignment, we aimed to gain introductory understanding by describing, interpreting, and analysing the athletes’ stories, which have been largely overlooked in academic research until recent years. We used one-to-one in-depth interviews to investigate female athletes’ lived experiences and perceptions, as well as the social processes and frameworks that influence their lives as athlete mothers in Norway. The athletes were selected from the Strong Mama project.^38^ The Strong Mama project’s primary aim was to explore the acute effects of performing high-intensity interval training (treadmill running, stationary bicycling, and heavy load resistance exercise) on maternal and fetal health in pregnant elite athletes and frequent exercisers. The secondary aim was to assess elite athletes’ experiences of pregnancy and return to sport, including maternal and fetal health aspects, sports performances, and practical challenges. To ensure the rigour of our research we strive to describe our method, data collection and analysis meticulously.

The inclusion criteria of the Strong Mama project, and consequently the present study, were athletes who were members of any national team or other high-level representative team in any sport organised by the National Sports Federation (e.g. the elite leagues in team sports such as handball and soccer) in Norway. In addition, we included athletes who were either actively pursuing or had intended to resume their sporting careers after pregnancy. We used a purposeful sampling method to identify suitable candidates. The selection process was a collaborative effort among the authors (MZM, EFMD, LFT, FEA, and LAHH). Candidates were chosen based on the variability of sports types, including group and individual sports, and the variability of supportive networks to ensure a diverse participant population with regard to work environments. We also aimed to select athletes who had both present and previous experience with pregnancy as well as diversity in parity, encompassing both nullipara and multipara. Including participants with various backgrounds and social contexts led us to retrieve information based on a larger group of athletes and not exclusively one profession or in one social context. All potential candidates were notified of the qualitative study by EFMD as a part of their involvement in the Strong Mama project, where they agreed to be contacted for further information. Six athletes were contacted by MZM through telephone or email and subsequently invited to participate in the study. One athlete declined due to lack of availability. Hence, five athletes were included in the present study; three athletes who were pregnant with their first child, one former athlete who had two children during her sporting career, and one athlete who returned to sports after having two children. All had recently participated in elite sports or intended to resume their sporting career after pregnancy (Table 1). One of the participants was born in Sweden, but lived and worked in Norway. To preserve confidentiality, limited information about the participants’ demographic is revealed.

**Table 1.** Participant description

Pseudonym	Age (years)	Social status/nationality	Parity	Sport
Hannah	31	Partnered Heterosexual Norwegian	Pregnant in third trimester Nulliparous	Team Ball Sports
Elle	29	Partnered Heterosexual Norwegian	Pregnant in third trimester Nulliparous	Individual Endurance Sports
Lisa	33	Partnered Heterosexual Norwegian	Pregnant in third trimester Nulliparous	Team Ball Sports
Mary	36	Partnered Heterosexual Norwegian	Had two children during her career Multiparous	Individual (Former) Athletics
Beth	29	Partnered Heterosexual Swedish	Returned to competition postpartum Multiparous	Team Ball Sports

Pregnant female athletes are a rare population with few informants in Norway, often less available to participate in research projects due to busy schedules and engagements abroad, and many of whom are contracted to international teams and living out of the country for parts of the year. Their insights are therefore valuable, as well as their time, leading us to focus on the quality of the interviews rather than a large participant selection. Based on the small pool of available athletes at this time, a sample of five participants was considered sufficient to provide meaningful information and enable a thorough analytical process.

### Researcher reflexivity

The present authors comprise five female (MZM, EFMD, LFT, KB, LAHH) and one male author (FEA), all currently based at the Norwegian School of Sports Sciences (NSSS) in Oslo, Norway. EFMD is the lead researcher of the Strong Mama study into pregnancy, health, training and motherhood in elite athletes.^38^ Her work in women’s health and implementation of the Strong Mama study served as the foundation for the present study. LFT, a professor in sociology, aided the study methodology with her extensive knowledge of qualitative research. FEA has experience in applied sport psychology and qualitative interviewing. KB is a trained physical therapist and a renowned exercise/sport scientist and LAHH has conducted extensive research on physical activity and health during pregnancy.

MZM, who conducted the interviews, works as a physical therapist in a clinical setting and has a master’s degree in Sports Physical Therapy from NSSS. While she has no formal background in qualitative interviewing, conducting personal interviews is an integral part of her work and was an asset when meeting with the athletes. She has firsthand experience of how women are treated in the healthcare system and the shortage of information on women’s health issues, prompting her to form the idea for the project. Collectively, the authors hold feminist views on equality in work and homelife and advocate for the advancement of maternity and postpartum care.

Researcher subjectivity is seen as a resource in thematic analysis.^39^ Balancing neutrality while striving to hold her own perspectives, MZM aimed to achieve meaningful and informative dialogue with the athletes. She asked open-ended questions, allowing the athlete to naturally guide the themes of the conversation derived from the interview guide. She followed tangents of conversation presented by the athletes, which she deemed poignant or thought-provoking. Acknowledging and displaying the authors’ unique experiences and viewpoints is essential to enhance reflexivity and transparency within research.

### Interview setting and data collection

The interview guide, initially developed by MZM, comprised 15 questions designed to guide the exploration of key conversation themes (Supplemental Material). Before finalising, it underwent multiple rounds of revision and refinement by the project group, including contributions and adjustments made by EFMD, LFT, FEA, and LAHH. The themes of the interview guide were based on a comprehensive literature review and the evidence summary from the IOC expert group regarding exercise and pregnancy in recreational and elite athletes.^40–42^ The interview guide was also inspired by the interview guide created by Davenport and colleagues^3^ with similar themes, but a slightly different line of questioning. Where Davenport et al^3^ questioned “what factors do you think limit elite athletes’ right to pregnancy?” we chose questions that were less assuming, such as “Can you say something about your own and other people’s expectations regarding pregnancy at the same time as a sports career?” Our wording was based on our limited knowledge of the climate and circumstances of elite athlete mothers in Norway.

Aligning with our study aim, the interview guide covered health, training, competition, and practical challenges. The latter included an open-ended question that asked the athletes to provide advice and recommendations to future athlete mothers based on their own experience. The interviewer (MZM) asked the athletes to reflect on the role that gender plays in their specific situation. Initially, this question was not included in the interview guide but was generated during the pilot interview. Given the interesting and thought-provoking viewpoints it garnered, we decided to include it at the end of all the personal interviews.

Based on the availability of the athletes, the interviews were conducted at different locations. These included a university campus, a sporting arena, a hotel lobby, and a parked car. Four interviews were conducted in person using a digital voice recorder and one was through video conference (Zoom Video Communications, Inc.).

The semi-structured interviews were conducted between October 2022 and April 2023, and ranged in duration from 32 to 72 minutes, with an average length of 51 minutes.

The interviews began with a short presentation of the interviewers’ background and motivations for the project as well as how the athletes’ contributions would be utilised. They read through the sheet of informed consent, whereby all athletes gave signed informed consent. A pilot interview of one participant was conducted to test the interview guide, estimate time required, and identify potential issues. With the participant’s consent, this data was included in the final dataset. One athlete was also contacted after the interview to record her responses to two questions that had been overlooked during the initial session. Research notes were made immediately post-interview noting the participant’s demeanour, the interviewer´s thoughts, and reflections during the interview. The notes were used to aid the initial transcription process.

### Data analysis

Thematic analysis, as described by Braun and Clarke,^43^ was used to analyse the collected material through six steps: (a) familiarising yourself with the data, (b) generating initial codes, (c) searching for themes, (d) reviewing themes, (e) defining and naming themes, and (f) producing the report. MZM transcribed the collected data and interview material using Microsoft Word. The interviews were transcribed verbatim and methodically checked for accuracy by listening to the audio recording several times and pausing frequently. Throughout this process, the material was de-identified to ensure confidentiality. Natural pauses or exclamations were retained in the transcript. The de-identified interview transcriptions were stored securely on MZM’s password-protected computer. The interviewer (MZM) read the transcripts several times, highlighting informative excerpts. She created descriptive codes signalling the core messages of the excerpts and sorted them using coding trees. This work was done manually on sheets of paper. Overarching themes emerged across the different interviews through revisiting and grouping codes. Recurring themes, relationships, and patterns, as well as specific and informative viewpoints and experiences, were identified for further analysis. The themes were later refined and relabelled through discussion with the research team and co-authors. Lastly, descriptive quotes reflecting the themes were extracted from the transcripts.

### Ethics statement

In accordance with the Helsinki Declaration (*The World Medical Association*, *2024*),^44^ the athletes were briefed orally regarding the project’s scope. Following ethical guidelines, they received written information in a leaflet detailing the project’s objectives and key interview themes. They were explicitly assured of their right to withdraw their interviews before publication, upholding autonomy and ethical integrity principles. To maintain confidentiality, the athletes were assigned pseudonyms. Only the authors viewed interview transcriptions, and specific information that could reveal the participant’s identity in the transcript was de-identified to maintain confidentiality. The athletes were informed that they could request a transcript of their interview and make corrections. The audio recording and both original and processed transcripts were kept in a password-protected personal computer. The Strong Mama project, including the present in-depth interviews, received approval on 12 September 2022 from the Regional Committees for Medical and Health Research (REK, reference number 478976) and the Norwegian Social Science Data Services (NSD, reference number 628051).

## Results

Through descriptive thematic analysis, our study uncovered five overarching themes related to pregnancy and motherhood in an athletic context: uncertainty, lack of supportive networks, physical capacity, the impact of postpartum return to sports on maternal health and combining motherhood and elite sports. The following text, supported by quotes from athletes, describes each of these themes, serving to illustrate and explain the athlete’s experiences and perspectives.

### Uncertainty

Uncertainty acted as a barrier to having children, which they attempted to mitigate by negotiating contractual maternity leave proactively. Lisa highlighted the importance of timing in family planning, comparing having children in her twenties as opposed to her thirties, and the implications this choice has on her career perspectives:

*“My disadvantage now is perhaps that I’m (in my thirties) and you start thinking about children and these things (…) wondering if they want me to continue (after pregnancy) because I’m getting old (…) as a 24-year-old you can have a long career afterward.”*

Some of the women operated abroad, where the policies regarding pregnancy leave and maternity payment differed from those in Norway. As a result, they had to negotiate contracts that ensured paid maternity leave and the continuation of their employment postpartum. Hannah said:

*“I had to sort of make an agreement with the club or tell the club when I started trying to get pregnant (…) then negotiate about salary while I’m pregnant so that I’d still have a salary.”*

The athletes described planning and coordinating their pregnancy to fall between major sporting events to avoid jeopardising contracts and future championships. They also expressed being nervous about how long it would take to get pregnant and if they were able to uphold their strict timeframe. Even if they managed to get pregnant in their desired timeframe, there was always uncertainty about whether they would successfully resume their careers postpartum. The unpredictability of bodily changes related to pregnancy and the postpartum period made for uncertain career trajectories. Elle explained:

*“You’re afraid of not coming back (…) but then you’re also very nervous about whether you’ll be able to get back in the same shape (…) I was really at the top of – maybe the best I’ve been (…) so it is very daunting to sort of leave a career that is going so well.”*

The key aspects of pregnancy planning were largely influenced by policy, contractual obligations, and economic situations, frequently leaving the athletes uncertain about their future in sports after starting a family. During pregnancy, the athletes tried to manage their training and trusted their instincts. Nonetheless, the lack of available guidelines on sports participation, including advice on high-intensity exercise, left them feeling uncertain. Mary explained:

*“Many times I’ve sort of thought like – is it safe, am I training too hard, is it the right thing to do, where am I crossing the line, am I harming the child?”*

Many of the athletes expressed a sense of isolation during their training routines, both during pregnancy and while returning to sports postpartum. They described uncertainty about their training practices and goals during these phases, frequently lacking accessible support networks to answer their questions. While some had sports colleagues who had undergone similar experiences, others found themselves pioneering this journey. Beth questioned: *“Who should I have asked?”.*

Hannah had doubts about the training she was doing, having little knowledge of when to moderate her efforts:

*“(If) someone had done a little more research or taken some tests during (my) pregnancy … I didn’t know if the kid was breathing or alive or if it is ok to run that fast or if I should slow down or if I could train even harder. So the uncertainty was sometimes a bit uncomfortable for me.”*

### Lack of supportive networks

The personal interviews showed varying degrees of support experienced by pregnant athletes. Some athletes benefited from robust support networks provided by their teams, fellow athletes, and the national Olympic Sports Center, while others navigated pregnancy on their own due to lack of supportive networks. They sought advice on their health, training during pregnancy, and the aim of returning to sport postpartum. Overall, their pregnancies were well received by their fellow athletes, and it speaks to the importance of fostering a supportive environment within elite sports communities. Hannah recalls:

*“With the national team, it has been just a bit like (…) that’s super great – congratulations and good luck and we hope to see you back.”*

Despite the coaching staff’s enthusiasm for Hannah’s pregnancy, they lacked experience in training pregnant athletes. She felt that both the coaching staff and her club avoided taking responsibility for her training practices:

*“They are very afraid of (…) influencing me and my training in any way (…) they were a bit hands-off.”*

The athletes distinguished between the feeling of being supported by their peers or team and what advice they received. Ultimately this led to a feeling of being cared for, but not actually helped or given adequate advice. The healthcare professionals the athletes consulted provided general advice on exercising during pregnancy. However, the athletes had to rely on their own judgment to determine when to stop or reduce the intensity of their training. Beth therefore thought the guidance she received fell short, which made her doubt the “professionals”’ expertise:

*“It didn’t feel like they had a lot of knowledge on pregnancy and elite sports.”*

Determining a safe level and intensity of training was often challenging, especially for first-time mothers. Elle experienced having Braxton Hicks contractions and was therefore advised to slow down or refrain from training. She found it hard to apply the healthcare professionals’ advice to her circumstances:

*“There were many (healthcare professionals) who sort of said – you can’t train if you have Braxton Hicks or you have to take it easy (…) I’ve had Braxton Hicks almost all the time regardless of whether I was calm or had a slow training session (…) it annoyed me because several gynaecologists only gave standard answers for the normal person (nonathlete).”*

Hannah searched for answers to her questions about maternal exercise online. She found that most of the information was geared towards the “normal” woman, emphasising moderate activity levels and relaxation.

*“It may be that there is something (information) out there that I don’t know about, but it’s certainly not easily accessible (…) it’s a bit of a shame. There is, after all, a small (research) gap in a way.”*

Mary trained and competed individually, without a team or coaching staff, which led to her receiving less information and advice. Because of this, she reached out to the relevant sports authorities and the Olympic centre for support. However, neither organisation provided the assistance she needed.

*“I was actually at the peak of my career and had a lot of sponsors (…) but I didn’t get that kind of support from the (Olympic Sports Centre).”*

She wondered how things might have turned out and how she would feel if she had received more information about prenatal and postnatal care, especially regarding her goal of returning to sport:

*“I was a bit pissed off afterward (after pregnancy) because I thought that if I had received support (…) if there was someone who had followed up on me (…) maybe the journey would have been different then?”*

Beth recalls resuming training only two weeks after giving birth. Reflecting on the experience, she believed she should have been more cautious. She also expressed a wish that someone from her coaching staff had offered her guidance during that time:

*“Nobody said anything (…) or just said ‘No, this isn’t too smart’ (…) when* *we were going to start sprinting (…) I faceplanted because my legs are still stuck in the ground while my upper body is racing forward.”*

While some of the women had already negotiated with their clubs before becoming pregnant, Beth found herself amid negotiations when she found out about her pregnancy. She decided to inform her club about the pregnancy before finalising and signing the contract, as a gesture of loyalty:

*“When I chose to tell (about my pregnancy) I didn’t hear anything more of it. Then I stood there (…) without a contract during my first pregnancy. You don’t have that support from the clubs.”*

Most of the athletes knew of other athletes in similar situations, prompting them to be meticulous when negotiating contract terms and planning for pregnancy.

### Physical capacity

When reflecting on their physical capacity during pregnancy the women agreed that they had more strength, power, and endurance than they had anticipated. Mary described her maternal physical capacity as a superpower:

*“Actually, you feel a bit like superwoman because you had something inside you (…) that made you a little stronger in some way.”*

The athletes faced few challenges in adhering to their training schedules during pregnancy. They maintained their frequency of training but adjusted intensity and dosage as necessary, avoiding unsafe situations like falls or collisions. Pelvic girdle pain was common, especially during the third trimester. The pelvic girdle pain retreated when they discontinued certain movements like sidestepping or running. They were astonished by their physical capacity during training. Some women felt stronger both during pregnancy and after giving birth, even setting personal records shortly after delivery. Lisa expressed:

*“I’m in great shape and I can train to the fullest (…) so far I have had an easy time being able to participate (in training during pregnancy).”*

Apart from pelvic girdle pain, the athletes experienced minimal pregnancy-related symptoms and no severe illness or pregnancy-related complications. Mary described her positive experience with her pregnancy:

*“I wasn’t ill during the pregnancy. I had a very nice pregnancy (…) everything came very easy to me, so I really just trained as normal.”*

The women also felt that having children could lead to more positive rather than negative impacts on their career development. Hannah explained:

*“I actually see it as something positive (…) many (athletes) are also in the best shape they’ve been after they have had children.”*

Several athletes talked about criticism regarding their exercise routines, mainly from other mothers, onlookers, and online communities. Mary perceived her training efforts as being negatively judged. She often received comments cautioning about her exercise habits and remarking on her choice to share training content on various platforms:

*“I also think it’s a bit sad that it’s not allowed to show that you’re strong, feeling well, functioning, having a nice pregnancy, and can come back quickly.”*

### The impact of postpartum return to sports on maternal health

Two athletes had experienced the postpartum period on several occasions. They both began training two weeks after giving birth and experienced considerable bodily changes during the first months postpartum. Both breastfed and aspired to return to sport as quickly as they managed. Mary experienced weight loss during breastfeeding but remained physically robust, achieving significant career milestones:

*“I lost weight rapidly so I got much skinnier (than before pregnancy) after I gave birth (…) I got comments like ‘oh my god you’re too skinny’ (…) or ‘do you have an eating disorder?’”*

Beth recalled entering the dressing room with her colleagues early postpartum and feeling uneasy about her changed physical appearance and body weight postpartum:

*“I thought it was awful coming back into the dressing room (…) to feel that I had a little bit extra (weight). I started training a lot (…) I lost way too much weight.”*

Despite feeling healthy after giving birth, Beth struggled with her postpartum physique, prompting her to engage in excessive training. She attributed her rapid weight loss to breastfeeding coupled with intense exercise regimes and sought assistance through an injury prevention hotline. A Dexa scan uncovered a previously undetected low muscle percentage.

The combination of breastfeeding and training played a significant role for both athletes, however, with very different outcomes. While it is complex to assume a causative association between breastfeeding, training, and postpartum weight loss, the notable weight reductions experienced by Beth and Mary had profound implications for their return to sport and overall health. This underscores the need for future research to understand how breastfeeding and training influence postpartum weight loss among athletes.

### Combining motherhood and elite sports

In the past, having a child while competing in elite sports often meant retirement, as few women had successfully returned to sports afterward. However, this trend has shifted in recent years. Beth recalled: *“I got so many questions like – ‘so you’ve retired already when you’re this young?’”*

Lisa pointed out that many athletes tend to postpone having children until their mid to late thirties, which often leads to the conclusion of their careers. However, by choosing to have children at the peak of her career, she finds it easier to resume training and competition afterward:

*“I think that it is career-extending to have children (…) It’s an incredibly nice way to also be able to keep doing what you love, for a long time.”*

Having children made her realise that there was more to her life than just her career:

*“(you get to be) a little more focused at practice and then there is something else, very lovely waiting at home.”*

Mary agreed that gaining new priorities and having to shift focus had positive effects on performance:

*“(previously) I could stay at (training) all day and just goof around (…) now I do what I need to and go home (…) I learned to discipline myself a little better.”*

Elle believed that there is a need for more stories of women who succeeded in having children during their careers:

*“You also need more people who choose to do it. There are some who do it, and it goes very well, but there are also a lot of people who can’t do it or give up. That’s probably most of them, even if you hear a lot of the positive stories.”*

All of the athletes had partners who were invested in sharing the parental duties, some of whom planned for the father to bear more of the responsibilities. They leaned on their partner to be the sole caregiver as they travelled to sporting events. The athletes recognised the importance of having a supportive partner, and they saw the division of parental duties as self-evident and unproblematic. However, some of the athletes were put off by unsolicited comments of how “lucky” they were to have such helpful partners, as mothers are often expected to be the “default” parent. They questioned whether similar expectations existed for male athletes. Some of the women had observed distinct societal expectations placed on male and female elite athlete parents. They felt that women were often held to a higher standard, particularly regarding household responsibilities. For instance, Lisa noted that many male athletes who had large families rarely faced questions about balancing work and home life, unlike their female counterparts:

*“There is a lot of expectation that it is the woman who should be at home, and it is the man who should get the chance to shine at work.”*

Hannah reflected on the demands that society, and especially the sporting world, put on mothers:

*“There is a mother and a father. Why is it the mother who must be the person who must sacrifice herself and her life? I don’t see how it can’t be a two-man job.”*

Travelling is a major part of an athlete’s life, often involving relocation to different countries and cities. Hannah described an expectation that partners of male athletes should relocate with them without hesitation. In contrast, when her partner followed her, he received praise, with many commenting on her luck in having such a supporting partner:

*“How many mothers are out there following their men (…) for example world champion players (…) all the men have wives and children. The wife lives there and they take care of the children and everything is fine. Why should there be such a big difference?”*

Hannah experienced that questions directed towards her were different from those aimed at her male colleagues. She often received questions on how she would be able to combine being a parent and an athlete.

*“No one asks … how he manages it (…) how he manages to be an elite athlete and a father?”*

## Discussion

The personal in-depth interviews revealed five key themes describing female athletes’ experiences and perspectives of pregnancy and motherhood in elite sport. The themes detailed uncertainty surrounding safe training during pregnancy and postpartum, combined with a lack of guidance from coaches and federations; the important role of support from partners in balancing motherhood and elite sports; the physical capacity, with some athletes reporting feeling stronger than ever before after giving birth; the impact of postpartum return to sports on maternal health, including struggles with body weight management while breastfeeding; and the mutual interplay between motherhood and elite sports, as athletes reported that the shift in priorities and focus introduced by motherhood had a positive effect on their performance.

### Uncertainty related to financial and contractual support

Our study highlights athletes’ financial concerns related to family planning. Consistent with prior research, financial instability presents a significant barrier to childbearing during a sports career.^3,4,20^ While Norway provides maternity leave and parental benefits and postpartum financial support for their residents,^45^ athletes on international contracts often depend on their income during pregnancy. Some of our athletes reported the need for family planning to be included in contract negotiations due to the existence of “anti-pregnancy” clauses that terminate contracts upon pregnancy.^20,46^ As revealed in the present study, one athlete even lost her contract after telling her club about her pregnancy, suggesting this could be a fairly common practice. As one athlete noted, the lack of adequate support often leads many athletes to end their careers prematurely. This highlights the need for governing bodies and sponsors to play a more active role in supporting female athletes who wish to start a family.^6,7,20,23,29^

### How supportive networks aid pregnant and postpartum athletes

Successful returns often hinge on comprehensive support systems, as described by previous qualitative research.^3,23^ Our athletes also described the challenges of training during pregnancy and postpartum without such networks, creating barriers in their journey to return to sport. They reported a lack of clear advice from healthcare professionals and coaches on training frequency, duration, and mode, highlighting a need to update cultural narratives surrounding training during pregnancy.

The supportive network within each athlete’s sports affiliation played a key role. Whether they had colleagues to lean on or a contact within the national Olympic Centre to guide them, impacted what information they received regarding training during pregnancy and postpartum. Athletes with teammates who successfully returned to sports after childbirth felt more confident in their decision to start a family. This echoes other qualitative research findings.^2,3,23,25^ Based on this, sports organisations may establish supportive networks for athletes – offering guidance and sharing of experiences about training during pregnancy and postpartum. A peer network of athletes who successfully returned to sports after childbirth, may foster an environment of confidence and informed decision-making.

### Pushing the boundaries of physical activity during pregnancy and postpartum

Our athletes maintained moderate to high-intensity training throughout their pregnancies, although with reduced volumes in the third trimester. This aligns with previous studies suggesting that elite athletes can modify their training patterns during pregnancy without substantially affecting their postpartum return to sports.^5,14^ Two of our athletes returned to sport just two weeks postpartum, and one reported setting personal records, demonstrating a surge of strength in her postpartum return. This is consistent with other recent studies suggesting that female athletes can maintain, and sometimes even improve, their performance post-pregnancy.^5,11,12,14^ However, not all athletes have such positive experiences. Kimber et al^18^ reported that 59 out of 150 athletes did not reach their athletic goals postpartum. Other studies have also reported that female athletes are more likely than male athletes to end their careers after having children.^7,22^ Given the knowledge of postpartum training, an individualised approach tailored to each athlete’s unique capacity and progress, could be the most effective strategy for achieving a successful return to sports.^19^

Our athletes suggested that sharing more success stories of athletes returning to sport after childbirth could inspire others and lessen the barriers to starting a family during an active sports career. These findings underscore the potential for viewing childbirth as a career enhancer rather than a career ender for elite athletes, provided they receive appropriate guidance and support.

### Postpartum return to sport and maternal health

Postpartum training and return to sports have largely been overlooked in research.^11,12,18,20,42^ While athletes previously relied on medical clearance to resume training six weeks postpartum, research now suggests that elite athletes with uncomplicated pregnancies can resume training shortly after giving birth, following graded activity principles.^12,23,42,47^

Training postpartum athletes, however, face an increased risk of injuries such as fractures and sprains, and issues related to pelvic health, compared to the average obstetric population.^10–12,18^ While causation is not yet determined, the athletes’ early return to sport and strenuous exercise regimes distinguishes them from the average obstetric population. It is speculated that hormonal changes during breastfeeding, which can cause laxity in joint capsules and connective tissues, may increase injury risk in postpartum athletes.^18^ Decreased bone mineral density has also been reported in athletes postpartum.^11^ Furthermore, lactation and breastfeeding could lead to relative energy deficiency in athletes.^24,35^

Two of our athletes who returned to sports two weeks postpartum experienced rapid weight loss, which impacted their return to sport and overall health. This weight loss was attributed to breastfeeding and vigorous training, with one athlete showing an alarmingly low muscle percentage on a Dexa scan. These athletes navigated their postpartum return to sport without professional guidance or a concrete rehabilitation plan of graded activity. Like the support provided during pregnancy, establishing peer networks to enhance knowledge could potentially aid athletes in their return to sport.^10^

Donnelly et al^19,47^ suggest an individualised, biopsychosocial approach with graded exercise progression for female runners. Furthermore, a recent consensus report on return to running postpartum^24^ concludes that the dosage of the exercise was more important than when an athlete returns to training, advising athletes to be conservative rather than “progress too fast too soon.” While recent studies aid in filling existing research gaps on training principles in the postpartum period, clear rehabilitation protocols for postpartum return to sports directed at elite athletes are still absent.^23,24,47^ Future research should aim to record the prevalence of postpartum injuries in elite athletes on a larger scale, establish causality, and propose concrete guidelines for training with the goal of returning to sport.^18,23,24^

### Combining motherhood and elite sports

While previous studies illustrate the pressure on women to conform to outdated gender ideologies that prioritise motherhood over career,^6,8,27–30,34^ our study found that athletes reject the notion that motherhood conflicts with their sporting identity. Historically, the athlete role has been associated with competitiveness, dominance, and aggression, while motherhood has been linked to selflessness and homemaking.^31,32^ However, our participants viewed their motherhood and sporting roles as complementary, and their pregnancy and the postpartum period as opportunities for personal growth and empowerment. They believed motherhood enhanced their resilience and adaptability, improving their performance in sports. Furthermore, sports participation helped them maintain their identity while allowing them to focus on their roles as mothers.

Our athletes’ rejection of traditional gender roles aligns with the egalitarian ideology of parenthood, which promotes work–life balance and equality among partners.^45,48^ This could explain the lack of guilt our athletes reported regarding balancing their sporting and parenting roles. Despite their confidence in juggling motherhood and sport, our participants doubted that male athletes faced the same pressures. Historically, men have pursued careers while women have managed both household and childcare duties.^49^ Even with supportive networks, women often bear the burden of domestic responsibilities, limiting their leisure time.^50^

In accordance with other qualitative studies,^8,27–30,34^ our data suggests a cultural change where women are progressively adopting provider roles while men are taking on caretaking responsibilities. Hence, the presence of a supportive family network can allow women to maintain successful careers while raising children, mirroring the support often provided to men.^22,51^

One of our athletes was tired of the societal narrative that placed her as the default homemaker, describing how her partner was held to a different parental standard. International elite athlete Serena Williams also openly addressed this cultural double standard in sport. As she retired from professional tennis, she commented,

*“Believe me, I never wanted to have to choose between tennis and a family. I don’t think it’s fair. If I were a guy, I wouldn’t be writing this because I’d be out there playing and winning while my wife was doing the physical labour of expanding our family.”*^52^

This statement illustrates how overt sexism still impacts elite athletes internationally and across sports.^27,29,30^

### Strengths, limitations, and methodological considerations

To our knowledge, this is one of two studies to explore the experiences and perspectives of Norwegian elite athletes regarding pregnancy and motherhood, using in-depth interviews. Hence, our study fills a critical gap in understanding these females’ multifaceted challenges in domains such as health management, training regimens, competitive pursuits, and logistical considerations of this region. Our findings may inform policymakers, clubs and federations to create lasting change within the athletic community. The findings also highlight the need for professional leagues and sporting clubs to have healthcare professionals well-versed in maternal healthcare as well as offer additional support in terms of family planning and childcare. Furthermore, our athletes’ positive experiences with training during pregnancy may inspire recreational athletes as well as the average obstetric population, to safely engage in regular physical activity throughout pregnancy.

The project’s methodology was based on existing accredited qualitative research and was thoroughly reviewed by the research group to promote validity. The five elite athletes were chosen for specific sports, career stages, and maternity phases (pregnancy, postpartum, etc.) to comprehensively capture female athletes’ experiences, enhancing transferability. Relying on one interviewer to conduct and transcribe all interviews helped promote dependability.

We chose to explore broad themes due to the lack of existing research on this population, and our comprehensive interview guide covered areas from pregnancy planning to return to sport, resulting in a wealth of data. As a result, we gained valuable insights into how athletes navigate these life challenges. Future research should aim to explore these topics individually and in detail, as they hold great potential as stand-alone topics.

Mirroring the population of the Scandinavian countries, our participant groups’ heritage, social situation, and frame of reference were fairly homogenous. Our findings may therefore be especially transferable to the Scandinavian region, but less transferable to other countries with dissimilar cultures.

Lastly, the qualitative research method is descriptive in nature, which allows the reader to get a glimpse into the lives of the athletes and their achievements and struggles, questioning the status quo and highlighting areas of improvement within elite sports in Norway. Our chosen method allows for an exploratory observation, consequently, further research could focus on more specific research scopes and variety within qualitative analytic approaches.

A limitation is that we conducted one interview via video conferencing, which was notably shorter, lasting 32 minutes compared to the others. Research indicates that face-to-face interviews typically yield more detailed responses.^53^ Direct translation might lead to loss of meaning or humour, idiomatic expressions, and cultural references stemming from the native language of this study. As MZM processed the five transcripts, her English vocabulary and diction may have influenced the translation quality.

Using a smaller sample of informants may have narrowed the diversity of viewpoints and experiences represented in our study. Hence, it is essential to acknowledge that our findings may not fully capture the perspectives of other relevant stakeholders in this context. By excluding sources such as coaches, partners, or healthcare professionals, we may have overlooked valuable insights that could have provided a broader context to our findings. Further research should strive to include a larger participant pool, as more and more high-profile elite athletes have become mothers since this project was completed.

## Conclusions

This study has investigated the experiences of female elite athletes as they navigate pregnancy and the postpartum period in the sporting world. Despite facing challenges, these athletes showed that they can successfully continue their sports participation throughout pregnancy and return to sports. However, more explicit policies are needed regarding pregnancy and maternity leave in elite sports, as well as further research on high-intensity training during pregnancy and safe return to sports postpartum. Our findings suggest that being a mother and an athlete can be complementary roles, and familial responsibilities can even enhance athletic goals. With the right support, motherhood could extend an athlete’s career rather than lead to early retirement.

## Supplementary Material

Supplemental Material: Interview Guide

## Data Availability

Due to the high-profile nature of the athletes in this study and the need to maintain their privacy, the raw data from the interviews will not be publicly available. However, the authors may provide anonymised excerpts or aggregated data upon reasonable and ethically approved request.

## Provenance statement

This article was not commissioned and went through external peer review.
