# Supplementary figures and images for: Experiences and perspectives on pregnancy and motherhood in elite athletes – a qualitative study
Source: Sex Reprod Health Matters. 2025 May 7;33(1):2501832. doi: 10.1080/26410397.2025.2501832 (PMC12120862; doi:10.1080/26410397.2025.2501832)

**Supplementary Materials**

**Interview Guide**


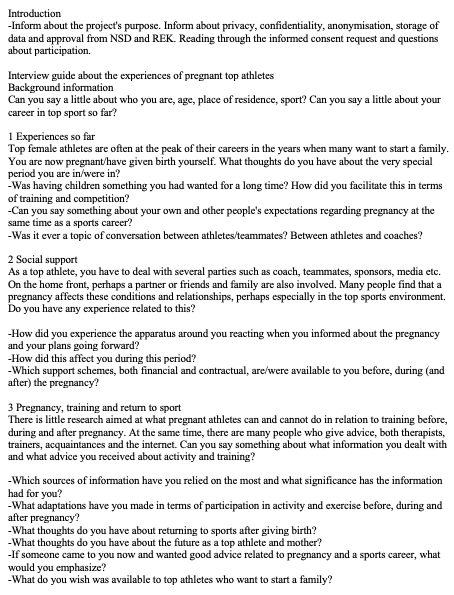

Supplement: Supplemental Material: Interview Guide [file ZRHM_A_2501832_SM0778.docx]
